# Are Supramodality and Cross-Modal Plasticity the Yin and Yang of Brain Development? From Blindness to Rehabilitation

**DOI:** 10.3389/fnsys.2016.00089

**Published:** 2016-11-08

**Authors:** Luca Cecchetti, Ron Kupers, Maurice Ptito, Pietro Pietrini, Emiliano Ricciardi

**Affiliations:** ^1^Department of Surgical, Medical, Molecular Pathology and Critical Care, University of PisaPisa, Italy; ^2^Clinical Psychology Branch, Pisa University HospitalPisa, Italy; ^3^BRAINlab, Department of Neuroscience and Pharmacology, Panum Institute, University of CopenhagenCopenhagen, Denmark; ^4^Department of Radiology and Biomedical Imaging, Yale UniversityNew Haven, CT, USA; ^5^Laboratory of Neuropsychiatry, Psychiatric Centre CopenhagenCopenhagen, Denmark; ^6^School of Optometry, Université de MontréalMontréal, QC, Canada; ^7^MOMILab, IMT School for Advanced Studies LuccaLucca, Italy

**Keywords:** rehabilitation, blindness, supramodal, crossmodal, sensory substitution, fMRI, MRI

## Abstract

Research in blind individuals has primarily focused for a long time on the brain plastic reorganization that occurs in early visual areas. Only more recently, scientists have developed innovative strategies to understand to what extent vision is truly a mandatory prerequisite for the brain’s fine morphological architecture to develop and function. As a whole, the studies conducted to date in sighted and congenitally blind individuals have provided ample evidence that several “visual” cortical areas develop independently from visual experience and do process information content regardless of the sensory modality through which a particular stimulus is conveyed: a property named *supramodality*. At the same time, lack of vision leads to a structural and functional reorganization within “visual” brain areas, a phenomenon known as *cross-modal plasticity*. Cross-modal recruitment of the occipital cortex in visually deprived individuals represents an adaptative compensatory mechanism that mediates processing of non-visual inputs. Supramodality and cross-modal plasticity appears to be the “yin and yang” of brain development: *supramodal* is what takes place *despite* the lack of vision, whereas *cross-modal* is what happens *because of* lack of vision. Here we provide a critical overview of the research in this field and discuss the implications that these novel findings have for the development of educative/rehabilitation approaches and sensory substitution devices (SSDs) in sensory-impaired individuals.

## Preamble

Over the past three decades, thanks to technological advances in sensory substitution (Bach-y-Rita et al., 1969) and functional brain imaging (Veraart et al., 1990; Sadato et al., 1996; Büchel et al., 1998), the study of the “human blind brain” presented neuroscientists with the opportunity to characterize the pivotal role of the (lack of) visual experience in forming a representation of the external world and in shaping brain development.

Sight has always been regarded as the most important sense for humans to interact with the outside world. Nonetheless, adults who are visually deprived since birth show perceptual, cognitive and social capacities that are often similar to those found in sighted individuals.

Historically, the blind brain has been primarily investigated from the perspective of the compensatory ability of early visual areas to process non-visual information (Sadato et al., 1996; for reviews see Frasnelli et al., 2011; Kupers and Ptito, 2011; Renier et al., 2014). At the same time, several experiments have been conducted to understand to what extent visual experience is a mandatory prerequisite for the human brain to develop its morphological and functional architecture (Ricciardi et al., 2014a). So far, several behavioral, structural and functional pieces of evidence have been collected in congenitally, early and late blind populations to characterize the distinct *cross-modal plastic* adjustments occurring after sensory deprivation on one hand, and the sensory-independent *supramodal* cortical organization on the other hand.

While supramodality and cross-modal plasticity often are thought of as being competing, mutually excluding explanations for the structural and functional organization in the blind brain, they are likely to represent “two sides of the same coin” or, to better underline their mutual interaction, the “yin and yang” of brain development. As a matter of fact, a great deal of the development of the brain architecture seems programmed to occur despite the absence of any visual experience, leading to a cortical organization able to process specific features of visual as well as of non-visual sensory information. At the same time, the lack of visual experience causes a cross-modal reorganization within portions of those brain areas that are deprived of their normal visual inputs, and start responding to non-visual stimuli.

As detailed below, the fact that brain areas may either respond to a specific information independently from the modality conveying the sensory input (i.e., supramodality) or adapt to respond to alternative non-visual inputs (i.e., cross-modal plasticity) represents the neural mechanism that should be taken into account for the appropriate planning of non-visual educational/rehabilitative programs or for shaping novel sensory-substitution devices (SSDs) in blind individuals.

## The Yin of Cross-Modal Plasticity

In cases of congenital absence or late-onset loss of sight, the deafferented subcortical and cortical structures, as well as their constitutive white matter tracts undergo substantial structural and functional reorganization (Ptito et al., 2008; Cecchetti et al., 2016; Reislev et al., 2016). These anatomical modifications are associated with the cross-modal functional recruitment of “visual” cortical areas during several non-visual perceptual (e.g., Watkins et al., 2013) and cognitive (e.g., Bedny et al., 2015) tasks. In addition, congenital, but not late, loss of sight is associated with an increased functional connectivity between primary auditory cortex and “visual” occipital regions, which relies on direct pathways (i.e., heteromodal connections), rather than on feedback inputs from associative brain areas (Collignon et al., 2013).

Interestingly, brain reorganization is not limited to cortical regions. Indeed, congenitally blind subjects encounter significant volumetric reductions of the whole thalamus, and particularly of the lateral geniculate nuclei. In sharp contrast, no volumetric changes were observed in the superior colliculus (Cecchetti et al., 2016). Consistently, congenital and early blind individuals, but not sighted controls, show a crossmodal recruitment of the “visual” midbrain (i.e., superior colliculus) during an auditory task (Coullon et al., 2015).

Early and prolonged lack of visual input leads to an adaptative reshaping of the brain that spreads beyond the visual areas. For instance, Noppeney et al. (2005) found an increase in the size of somatosensory and motor white matter fibers in early blind subjects, whereas others reported a thickening of the cingulate and frontal cortical areas, together with a thinning of the somatosensory and auditory cortex (Park et al., 2009). On the other hand, functional studies revealed a substantial reorganization within primary “non-visual” cortices of blind subjects, such as an expansion of the cochleotopic portion of the auditory cortex (Elbert et al., 2002) and enlarged somatotopic representation of the fingers in multifinger Braille blind readers (Sterr et al., 1998). This form of “intramodal” plasticity may depend on the multisensory tuning that occurs during development and that is shaped by specific perceptual learning and experience (Proulx et al., 2014).

Although a significant number of studies have investigated which mechanisms drive the crossmodal reorganization in the blind brain and to what extent its plastic reshaping has functional and behavioral advantages, an unequivocal answer to these questions is not yet available. For instance, if volumetric properties of the occipital lobe can predict behavioral accuracies in pitch discrimination (Voss and Zatorre, 2012), or if the recruitment of “visual” cortex during Braille reading is modulated by blindness onset (Burton et al., 2002), correlations between performance and crossmodal recruitment of deafferented cortical areas has also been demonstrated in a variety of other tasks, such as olfactory (Renier et al., 2013), auditory (Ross et al., 2003; Voss et al., 2008; Renier et al., 2010) and tactile (Kupers et al., 2006).

## The Yang of a “Supramodal Mechanism”

There is now ample evidence that the development of the morphological and functional architecture of the human brain is to a large extent independent from visual experience (Pietrini et al., 2004; Ricciardi and Pietrini, 2011; Ricciardi et al., 2014a,b,c). *Supramodal* (or *metamodal*, with a Latin or a Greek root, respectively) responses do not depend on a specific sensory modality, but rather on the distinct content to respond. Some authors therefore refer to “task-specific sensory-independent” activity (e.g., Heimler et al., 2015) to indicate how supramodal brain areas respond to a given perceptual information or task, independently from the sensory modality that conveys the input to the brain.

Supramodal processing within the “visual” extrastriate system has been studied in both sighted and congenitally blind individuals. In particular, research has been conducted on form recognition, motion discrimination, spatial and navigational processing, using visual and non-visual sensory tasks in both congenitally blind and sighted individuals (e.g., Sathian et al., 1997; Zangaladze et al., 1999; Amedi et al., 2001; Hagen et al., 2002; James et al., 2002; Merabet et al., 2004; Pietrini et al., 2004; Cate et al., 2009; Kitada et al., 2009, 2014). These studies have demonstrated that neural responses in “visual” areas during non-visual processing are not merely related to visual imagery, and that visual experience is not a mandatory prerequisite for the functional specialization within the visual system (Pietrini et al., 2004; for a review see Ricciardi and Pietrini, 2011).

The fact that specialized subregions of the “visual” system are supramodally recruited has been confirmed using several protocols that conveyed the same information (i.e., shape form, spatial layout, etc.) across different non-visual sensory modalities and demonstrated overlapping neural responses in both sighted and blind samples. Equally, sensory-independent responses can be impaired by transcranial magnetic stimulation (TMS)-induced lesions in task-specific “visual” areas (e.g., Noppeney, 2007 ; Collignon et al., 2011; Frasnelli et al., 2011; Kupers and Ptito, 2011; Kupers et al., 2011). More recently, the employment of multivariate pattern recognition approaches offered a novel tool to demonstrate a shared coding of specific stimulus content, such as shape, motion and action, in both sighted and congenitally blind individuals across different sensory modalities (Pietrini et al., 2004; Mahon et al., 2009; Ricciardi et al., 2013; Dormal et al., 2016; Handjaras et al., 2016). Noteworthy, the homologies in the neural patterns of stimulus representation obtained with multivariate approaches are not typically limited to a mere overlap in the spatial localization of “activated” regions, but actually do involve the intrinsic content of the neural responses, suggesting that sensory-independent representations are somehow (hard)-coded at a neural level (Ricciardi et al., 2013; Handjaras et al., 2016).

## What Did We Learn from Sensory-Substitution Studies?

Recent studies using SSDs also support the concept of supramodality. An SSD typically converts visual into non-visual information, and relies on the response of the same brain region that would have selectively processed that “specific visual information”. Consequently, the sensory content provided through SSDs is processed in a task-specific manner by supramodal cortical areas both in sighted and blind individuals. For instance, SSDs that translate “what” (i.e., shape) and “where” (i.e., location) properties of a visual stimulus into auditory information recruit the ventral and dorsal visual pathways in congenitally blind people, respectively (Striem-Amit et al., 2012b; see also Ptito et al., 2012).

Within the extrastriate “visual” cortex, SSDs recruit functional modules tuned to process motion, body-parts and shape information. The motion-sensitive middle temporal cortex (hMT+) is recruited by motion information conveyed by a visual-to-tactile SSD (VTSSD) in sighted and in congenitally blind individuals (Matteau et al., 2010). Similarly, perception of body shapes through a sensory-substitution algorithm in blind subjects is mediated by recruitment of the extrastriate body area (EBA; Striem-Amit and Amedi, 2014). Likewise, a portion of the lateral occipital complex (LOtv) is activated in a shape recognition task using a visual-to-auditory (VASSD) or a VTSSD (Amedi et al., 2007; Ptito et al., 2012). Blind individuals can even process shape and color features by means of SSD-generated auditory stimuli (Abboud et al., 2014). Also, blind individuals recruit the visual word form area (vWFA), a specific brain region that is thought to process the visual representation of letters, when reading through a visual-to-auditory SSD (Striem-Amit et al., 2012a). Of note, the observation that VWFA is also recruited in blind individuals via tactile recognition (Reich et al., 2011) and by sighted subjects during Braille reading (Siuda-Krzywicka et al., 2016), along with the predetermined cortico-cortical wiring of this region with superior temporal and inferior frontal regions in preschoolers (Saygin et al., 2016) confirms the hypothesis of modality-independent processing of information in supramodal regions.

**Figure 1:**
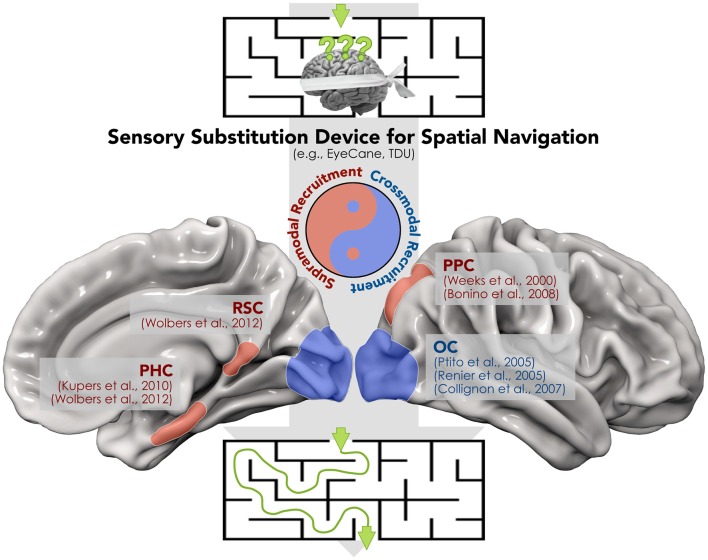
**A proof of concept for the synergistic interplay between crossmodal and supramodal brain functioning during the spatial navigation task carried out by means of a sensory substitution device (SSD).** The recruitment of both modality-independent brain regions within the “spatial navigation network” (i.e., retrosplenial (RSC), parahippocampal (PHC) and posterior parietal cortex (PPC)) and the crossmodal activation of “visual” cortices in blind individuals contribute to avoidance of obstacles and the identification of the correct route.

SSDs have been also employed in blind individuals during more complex tasks such as spatial navigation (Kupers et al., 2010; Chebat et al., 2011, 2015; Proulx et al., 2015; for a review). The ability to navigate the environment is crucial in modern urban life, yet it represents a challenging task for blind subjects, in particular when novel routes have to be learned. In addition, spatial navigation strategies differ between congenitally blind and sighted subjects, since the former rely more on egocentric than allocentric coordinates (Pasqualotto and Proulx, 2012; Pasqualotto et al., 2013). Using a VTSSD (tongue display unit—TDU; Bach-y-Rita, 2004), Chebat et al. (2011) demonstrated that congenitally blind individuals are able to detect and avoid obstacles during a spatial navigation task. The ability of visually-deprived individuals to detect and avoid obstacles has been confirmed in a more recent study using the EyeCane, a VASSD (Maidenbaum et al., 2014). Indeed, after a brief training with the EyeCane, congenitally and late blind subjects demonstrated a number of collisions and time to complete a virtual and a real life-size maze, similar to sighted participants with no blindfold (Chebat et al., 2015). For a proper and autonomous interaction with the surrounding space, the capability to follow a specific route and avoid obstacles should also be associated with an active tracking and reaching of objects. The latter abilities have been tested in blindfolded sighted subjects while using EyeMusic (Abboud et al., 2014), a VASSD that translates the spatial location of a target into the pitch of musical notes. Levy-Tzedek et al. (2012) showed that using EyeMusic, participants performed fast and accurate movements similar to those carried out with visual feedback. Kupers et al. (2010) used fMRI to examine the cerebral correlates of navigation in the absence of vision. These authors reported that congenitally blind subjects recruit the parahippocampal cortex (PHC) during TDU-guided spatial navigation, the same area that is activated when sighted individuals perform the same spatial navigation task under full vision. In addition, several other brain regions that are supramodal in nature (Weeks et al., 2000; Ricciardi et al., 2006; Bonino et al., 2008; Wolbers et al., 2011) and are involved in spatial localization and representation, such as the posterior parietal (PPC) and retrosplenial (RSC) cortices, were activated (Figure 1). Finally, a recent report supported the idea that different sensory modalities can produce very similar spatial representations (i.e., supramodal) through SSDs in sighted subjects (Pasqualotto and Esenkaya, 2016). Taken together, the above data suggest that the recruitment of these regions through SSD depends on sensory-independent task-related activity, which encodes a more abstract representation of information content.

On the other hand, stimulation protocols via SSDs provided also a strong support to crossmodal plasticity. Therefore, it should not be surprising that most of these SSD-mediated protocols reported activations in the occipital cortex in blind individuals during the use of VASSDs and VTSSDs. For instance, using positron emission tomography (PET), Ptito et al. (2005) demonstrated recruitment of the occipital cortex after a brief training with TDU for congenitally blind individuals, but not for blindfolded sighted controls (Figure 1). The recruitment of occipital regions in blind participants was confirmed by a later TMS experiment from the same group (Kupers et al., 2006). In this study, it was shown that stimulation of the occipital lobe produced tactile sensations of the tongue in blind individuals who were proficient with the TDU. The evidence for a similar cross-modal recruitment has been reported in studies using VASSD in blind subjects (Arno et al., 2001; Collignon et al., 2007; Merabet et al., 2009), and even in sighted participants after training (Renier et al., 2005). In addition, a more recent report suggests that occipital responses induced by SSD in blind individuals are primarily driven by top-down connectivity, i.e., by a specific task rather than a specific sensory channel, and are modulated by blindness duration (Murphy et al., 2016).

These findings suggest that the recruitment of the occipital cortex in proficient blind SSD users, may be mediated by the “unmasking” or strengthening of pre-existing connections (Kupers et al., 2011).

## A Contribution to Visual Rehabilitation and Future Challenges

Several findings indicate that the topographic organization of the brain is largely preserved in congenital blindness, and that distinct cortical areas are able to process information independently from the sensory modality that carries that content to the brain. This supramodal organization is a genuine intrinsic characteristic of the brain, as it is also present in sighted individuals. This has important implications not only for the understanding of how the brain works, but also for how blind individuals form a mental representation of the external world. Indeed, the more abstract nature of mental representations in the brain accounts for the ability of congenitally blind individuals to acquire knowledge and interact efficiently with a world that they have never seen. Thus, the blind brain should not be considered as a “disabled”, but as “differentially abled” brain. Therefore, a “sensory isolation” of visually-deprived individuals, by reducing or limiting the exposure to perceptual, cognitive or social experiences, would likely be one of the worst “educational” choices.

As above-mentioned, the specific content of information could be conveyed through non-visual sensory modalities. More importantly, supramodal organization and crossmodal plasticity following lack of vision may both contribute to the rapid adaptation when using SSDs. On the other hand, the relationship between the proficiency in performing a specific task through the use of SSDs and the crossmodal plastic phenomena described in blind individuals is still to be fully exploited, as some authors found no behavioral differences between sighted and blind individuals (Abboud et al., 2014; Maidenbaum et al., 2014).

From an epidemiologic perspective, it should be pointed out that the increase of life expectancy in Western societies has led to an increase in the number of visual impairments due to chronic eye diseases and aging (World Health Organization, 2007). In light of this, the proportion of people losing sight at later stages of life is growing and the study of rehabilitation protocols tailored to meet the needs of “late-blind” individuals are assuming more and more socioeconomic relevance. The research on rehabilitation and neuroprosthetic tools should seriously account for this. In particular, some authors reported that the degree of compensatory changes following loss of sight is influenced by the age of blindness onset and is reflected by the extent of cross-modal recruitment within “visual” occipital areas (Voss et al., 2008; Bedny et al., 2012; Collignon et al., 2013). Thus, late blind individuals could provide a fundamental model to exploit the potential of SSDs in sighted individuals who lost vision later in their lives.

Which kind of information should future SSDs convey? On the one hand, recent studies demonstrated that object recognition through VASSD is affected by capacity and resolution limitations related to the processing of auditory stimuli (Brown et al., 2014; Brown and Proulx, 2016). On the other hand, the description of supramodal responses recently moved from simpler perceptual to more cognitive stimuli, such as actions or events, to emotion and social functioning (Bedny et al., 2009; Ricciardi et al., 2009; Klinge et al., 2010; Mahon et al., 2010). These more complex cognitive tasks rely on distributed brain networks, and are not limited to functionally specialized cortical clusters involved in processing simple sensory features of stimuli. Consequently, at which level (e.g., localized area or network) and how does the supramodal representation of information occur for more complex cognitive tasks? We recently demonstrated that circumscribed brain areas retain a modality-dependent processing of simple unisensory information, whereas larger networks are able to integrate the semantic content of sensory information and to generate a modality-independent representation that matches language and retains the most precise definition of concepts (Handjaras et al., 2016). This supramodal mechanism of distinct levels of stimulus processing may explain how information progresses from a sensory-based towards a more abstract conceptual representation (Mahon and Caramazza, 2011; Ricciardi and Pietrini, 2011; Ricciardi et al., 2013; Handjaras et al., 2016). From a translational perspective, we can ask how rehabilitative approaches or SSDs (which typically dissect and reproduce definite spatio-temporal features of sensory stimuli) will evolve from processing simple sensory information to processing more complex stimuli, including emotional and affective ones. This would indeed represent a major challenge for future translational research.

To conclude, rehabilitation in visually-deprived individuals should be considered as a complex educational and learning process. Rehabilitation is not limited to the “simple” acquisition of a perceptual/cognitive strategy or of the skills needed to utilize an external aid. An innovative and proper rehabilitation strategy comprehends several intimate and socio-environmental aspects of the blind individual—particularly in late-onset blindness—that aim at an autonomous and efficient interacting with the surrounding world.

## Author Contributions

LC, RK, MP, PP and ER contributed to the conception of the work; LC, RK, MP, PP and ER drafted the manuscript; LC, RK, MP, PP and ER critically revised the manuscript; All the authors approved the final version of the manuscript.

## Conflict of Interest Statement

The authors declare that the research was conducted in the absence of any commercial or financial relationships that could be construed as a potential conflict of interest.
